# Eukaryotic Cell Membranes: Structure, Composition, Research Methods and Computational Modelling

**DOI:** 10.3390/ijms241311226

**Published:** 2023-07-07

**Authors:** Anatoly Zhukov, Valery Popov

**Affiliations:** Timiryazev Institute of Plant Physiology, Russian Academy of Sciences, Botanicheskaya Street 35, 127276 Moscow, Russia; zhukov_anatoly@list.ru

**Keywords:** membranes, lateral heterogeneity, phospholipids, glycolipids, integral membrane proteins, lipid rafts, nanodomains, computer modelling

## Abstract

This paper deals with the problems encountered in the study of eukaryotic cell membranes. A discussion on the structure and composition of membranes, lateral heterogeneity of membranes, lipid raft formation, and involvement of actin and cytoskeleton networks in the maintenance of membrane structure is included. Modern methods for the study of membranes and their constituent domains are discussed. Various simplified models of biomembranes and lipid rafts are presented. Computer modelling is considered as one of the most important methods. This is stated that from the study of the plasma membrane structure, it is desirable to proceed to the diverse membranes of all organelles of the cell. The qualitative composition and molar content of individual classes of polar lipids, free sterols and proteins in each of these membranes must be considered. A program to create an open access electronic database including results obtained from the membrane modelling of individual cell organelles and the key sites of the membranes, as well as models of individual molecules composing the membranes, has been proposed.

## 1. Introduction

Biological membranes are similar to the structures of inheritance, such as nucleic acids, from the point of their importance in the living world; they may have evolved even earlier than the nucleic acids. Biological membranes are difficult to explore, both because of their molecular profile and structure, and the other reason is that they function over a wide interval of time scales [1]. The functional diversity of cell membranes includes multiscale patterning and the formation of a range of forms that vary from local organizations of nanometre-scale lipid–protein domains to micrometre-scale organelles with architectures of planar, curved and tubular structures. The basic structure of cell membranes is a bilayer composed of two layers of polar lipid (PL) molecules, into which proteins with crucial functions, such as enzymes, receptors and transporters, are partially or completely embedded [2,3].

The cells of all eukaryotic organisms, including plants, in addition to being restricted by a membrane themselves, are divided into various compartments which are also encircled by membranes; these entities are called organelles. The membranes represent the main cell structure of all eukaryotes—their soft skeleton. Many important metabolic processes occur on membranes, since the other main component of all eukaryotic cells—numerous enzyme systems—is essentially membrane-bound; often the enzymes are not able to perform their functions outside of these membranes. Another crucial element of plant cells—photosynthetic pigments and the reaction centres where these pigments function (photosystems I and II)—needs to be strictly organised structurally. These reaction centres are known to be localised in the membranes of stromal and granular thylakoids [4]. Finally, the entry of pathogenic viruses into living cells also takes place through the membranes. The entire membrane is known to be 7–8 nm thick, whereas the corresponding value for its internal hydrophobic layer is assumed to be as little as 3 nm [5]. 

The basic constituents of cell membranes are PL, which exist in an aqueous medium of cells as vast bimolecular layers, having a hydrophilic external surface and a hydrophobic internal space. Similar to bilayer-permeating protein molecules (integrin and transmembrane proteins), they form the basis of the liquid mosaic structure of biological membranes and specify their function [6]. The diversity of profile of membrane lipids between species and the distinction of their contents are strictly linked to the multifaceted role which the membranes play in plant organs [7]. Apart from lipids, biomembranes (in particular, the plasmalemma) are composed (up to 80% by mass) of proteins, free sterols and other non-lipid elements, which together define the specificity and wide range of membrane biological activities. The components also include carbohydrates, which, however, are not represented by the separate compounds, but constitute a part of glycolipids or protein molecules [8,9,10].

The classic model of biomembranes for 50 years has been the liquid mosaic model of Singer and Nicolson [11]. It suggests that the lipid bilayer of eukaryotic membranes is an unstructured liquid characterised by a disorderly and chaotic arrangement of lipid and protein molecules [12]. The model was continually improved over the following decades. Many reviews trace a chronology for key advances in this field [13,14,15,16,17,18]. Significant advances have been made in the study of eukaryotic cell membranes, especially with regard to the plasma membrane (PM) [19,20,21,22]. The presence of the Singer and Nicolson model, apart from its undisputable usefulness, has often been a noticeable obstacle for further research. It has been commonly assumed that all the other membranes in many different organisms are structured according to PM principles [12,23]. Therefore, the diverse membranes of many cell organelles of all eukaryotes and, in particular, those of plant cells have not been widely studied [24,25,26,27,28,29,30]. In 2014, Nicolson modified the original model, naming it the “biological membrane structure model with a liquid mosaic” [13]. It has been shown that cell membranes consist of a complex mosaic of many microdomains containing different clusters of proteins and lipids, which are distributed in a vast area of a thin membrane layer [23,31,32,33,34]. 

The study of membranes has led to the discovery not only of their domains but also of the individual varieties of the domains, namely lipid rafts [9,35,36,37,38,39,40]. In 1997, Simons and Ikonen [41] suggested a concept of a ‘lipid raft’ to explain the lateral irregularity of the membrane; a lipid raft was described as a small (10–200 nm), heterogeneous, hugely dynamic region abundant in sterols, glyco- and phospho-sphingolipids and phospholipids (PhL) with saturated fatty acids (FA) [42]. Lipid rafts are characterised as microdomains of the lipid bilayer of the cell membrane, where areas with a higher packing density of lipid molecules are formed around particular proteins [43,44]. The functions of the cytoskeleton and glycocalyx have been subsequently outlined in the more complex membrane models [18]. Cholesterol (CHOL) [32,42] and the phosphosphingolipid sphingomyelin (SM) [10,45,46] in the case of animal tissues, and sitosterol or stigmasterol as well as glycosphingolipids in plant tissues are recognized as the two major components of lipid rafts [9,47,48,49]. The role of glycerophospholipids (GPhL) as a possible element of lipid rafts has not been given sufficient attention yet [42,50,51,52].

The opportunities of optical microscopy allow the detection of membrane areas with sizes of 200 nm or more; so, lipid domains or rafts, which are generally smaller, are not distinguished under the optical microscope [53,54]. The creation and study of simplified membrane models are helpful in this way, making it possible to control numerous factors that occur in membranes in vivo [2,55,56]. Moreover, computer modelling, which could be named a “computational microscope”, is actively used [1,57,58,59].

The composition of polar lipids (PL) is known to vary greatly in different cell organelles and also with respect to the internal and external membrane layers [2,5,9,26,60,61]; this is reflected in the structure and features of membranes. It would be appropriate to study each individual membrane based on the molar content of all its components and to construct a model of the membrane either in 2D or in 3D. A suggestion could be made to register the findings not only in scientific articles, but also using some kind of common platform. Thus, it would be very useful to create a database and a kind of “biomembrane encyclopaedia”, similar to what has already been created for genes and the genome, called KEGG (Kyoto Encyclopaedia of Genes and Genomes). Different models of individual cell organelles or their characteristic regions should be collected and represented in this database, as far as possible, within the range from 200 nm to 0.5 nm. Models of artificial membranes may also be represented in this database. This database should also include modelling data for individual molecules of compounds making up membranes, such as PL, sterols, proteins, etc. Most importantly, the data will be concentrated in one place and be easily accessible. We believe that the creation of this platform will advance the study of the membranes of all eukaryotes.

## 2. Structure and Functions of Membranes in Various Eukaryotic Cell Organelles

It is essential to have an understanding of the functioning of individual organelles before studying the structure of cell membranes. It is obvious that PM is not the standard for the structure of all the other membranes. Membranes of eukaryotic organisms separate the cell into individual subcellular compartments that carry out crucial, while often non-compatible, metabolic reactions. The organelles are also differentiated both quantitatively and qualitatively in terms of lipid content. The membranes of chloroplasts and mitochondria, for example, have a lipid composition that differs sharply from that of PM. Animal tissues differ from plant tissues in PL composition, and the internal and external membranes of the two-membrane organelles are also different. The lipid profile of the external and internal layers of the membrane bilayer varies considerably in many organelles, resulting in an asymmetry throughout the whole bilayer; the situation is maintained by ATP-dependent flippases [2,5].

Eukaryotes have developed membrane compartmentalization to allow for the formation of enclosed environments for specific metabolic processes. At the same time, they have created effective transport mechanisms in order to facilitate their metabolism via membrane bilayers [62]. All cell organelles can be either bimembranous, like the nucleus, mitochondria and plastids, or unimembranous, like the endoplasmic reticulum (ER), Golgi apparatus, vacuoles and peroxisomes.

At present, the plasma membrane (PM) is the most studied bilayer. The PM is a complex system composed of the two layers of lipids and proteins, which are further subdivided into small units called nanodomains. While both layers are asymmetrical, the bond between them is remarkably strong. For instance, this can be demonstrated by experiments carried out with giant phospholipid single-layer vesicles; these revealed that the nanodomains formed in the external layer coincide perfectly with those in the internal leaflet. Likewise, the microscopic separation of phases in one leaflet may cause the separation of phases in the opposite leaflet, which would otherwise be homogeneous [63].

The lipid composition of PM varies considerably among various organisms and cell types; it is also affected by the stage of the cell cycle as well as by environmental factors. Usually an average composition of an idealised mammalian PM is taken as an example, with sphingomyelin (SM), phosphatidylcholines (PC) and gangliosides mainly in the outer layer, and phosphatidylethanolamines (PE), phosphatidylserines (PS) and other charged lipids in the internal layer of the membrane. Bound lipid FA vary from fully saturated to polyunsaturated, with a large proportion of unsaturated FA belonging to the inner lipid layer. Eukaryotic PMs also contain 20–50% of free sterols [64].

The plant cell vacuole membrane, named a tonoplast, seems much less explored. The tonoplast has a selective permeability; it actively participates in the transport of metabolites required for cellular osmoregulation, the regulation of cytosolic pH and ion balance, and the transduction of signals of various origins; the tonoplast is also actively involved in protecting cells against abiotic stress. The majority of these functions are strongly associated with tonoplast lipids [28]. The vacuolar membrane determines the specificity of plant cells; it is actively involved in the intracellular redistribution of substances and also acts similarly to lysosomes. The tonoplast has an ontogenetic link to the ER and the Golgi complex. It is generally accepted that mature central vacuoles are giant lysosomes of plant cells. Water-soluble substances are able to be transferred to the vacuole by small membrane vesicles, which, being fused to the vacuole, simultaneously carry out its growth and also regulate the composition of the vacuolar contents. The tonoplast prevents the leakage of a number of metabolic poisons and acidic hydrolases into the cytoplasm, thereby keeping the plant cell viable.

The bilayer nuclear membrane is the best example of where the efficient transport mechanism occurs; it forms a physical barrier to segregate genomic DNA and isolates transcription and translation. This envelope contains multitudinous holes called nuclear pores, consisting of the largest protein complex in cells, known as the nuclear pore complex. The complex contains 500–1000 proteins (nucleoporins) of up to 40 different species that assemble in a highly conserved channel, allowing the free movement of molecules smaller than 40 kDa and, what is more important, mediating the selective relocation of larger molecules and molecular complexes between the nucleus and cytosol [62].

ER membranes deserve special consideration as they are not only composed of PL themselves but are also the site of synthesis of both PL and their constituent FA. The ER is a continuous network of tubules consisting of lamellar and tubular cisterns which permeate the entire cytoplasm and connect to the nuclear envelope. The membranes of these tubules account for over half of the total number of all cell membranes. The inner cavity of the ER is isolated from the cytosol by a single membrane only; this cavity of ER occupies at least 10% of the total cell volume. The ER membrane has multiple folds and curves and forms a continuous surface, restricting a unified internal space (cavity) [65]. It should be noted that the fractioning of destroyed cell organelles by centrifugation leaves ER fragments in the microsomal fraction.

The ER is characterised by an almost immovable, fixed arrangement of spiral-shaped network sections and tubules with blind endings. The initial ER structure is tube-shaped tubules characteristic of smooth ER. Rough ER diverges from smooth ER by the presence of ribosomes attached to its membranes. One of the key functions of smooth ER membranes is the synthesis of lipid molecules, so that they are enriched with various enzymes for the biosynthesis of both glycerolipids and other closely related components. The remodelling of the plant ER probably has a value for its function in targeting protein secretion, organelle interactions and signal exchange. The structure and mobility of the ER are primarily regulated by the actin cytoskeleton via actin motor proteins and membrane-cytoskeleton adaptors. Recent discoveries have also detected alternative pathways that affect ER movement through a microtubule-based mechanism. The ER has a continuous membrane-enclosed network of leaf-like (cisterns) and tubular structures that are closely linked to the cytoskeleton. The membranous ER tubules continuously expand, contract and merge with each other, and the polygonal structures vary dynamically due to changes in tubule branching patterns and the expansion of the ER cisterns [30].

Mitochondria are generally spherical or rod-shaped organelles 1–3 μm long and about 0.5 μm in diameter; each cell contains several hundred of them. Their small diameter makes them difficult to observe via light microscopy; it delays their detection [29]. Mitochondria in a living cell move continuously by means of cytoplasmic streaming linked to the cytoskeleton. Under certain conditions, these organelles can merge forming large, reticulated mitochondria. The outer mitochondrial membrane is isolated from the inner mitochondrial membrane by a less electron-dense zone called the inter-membrane space. The inner membrane surrounds the electron-dense matrix, but it also creates invaginations, called cristae, in the matrix area. The outer membrane has pores with a diameter of 2.8 nm, which are anion channels [29].

A biochemical analysis of the internal membranes of chloroplasts showed that the ratio of lipids to proteins was close to 1:1 by mass while their molar ratio was 500:1. Chlorophylls, carotenoids, plasto- and phylloquinones that make up about 65% of the lipids in these membranes are classified as lipids here. Moreover, chloroplast membranes contain a lot of α-tocopherol, which increases their tolerance to free-radical processes. Sterols provide rigidity to membranes by occupying the free area between the hydrophobic tails of lipids and ordering their arrangement [41,66]. The internal membrane of the chloroplast envelope is involved in the creation of the inner membrane system of these organelles. The internal membranes form thylakoids, which are stacked (granulated to grana thylakoids) or permeate the stroma, connecting the grana to each other (stroma thylakoids). Accordingly, their forming membranes are referred to either as grana or stroma membranes. Thylakoids of the grana may be permeated by one or more thylakoids of the stroma.

Peroxisomes are multiple organelles surrounded by a single bilayer membrane, lacking DNA; they have a diameter of 0.1–1.5 μm. Despite their small diameter and simple structure, peroxisomes are very morphologically and metabolically dynamic structures and they play an important role in the metabolism of animals and plants. These organelles are very diverse in morphology and protein content when responding to various developmental and environmental signals. In plants, peroxisomes are important for growth and development; they execute a variety of metabolic functions, many of which are coordinated with other organelles such as mitochondria, chloroplasts and oleosomes, through their physical interactions [67].

In both plant and animal cells, the Golgi apparatus is a series of individual stacks of disc-shaped membrane cisterns; this series is known as dictyosomes. The cisterns are continuously joined by membrane vesicles (vesicles) budding from the granular ER [65]. Liposomes are a unique type of polymolecular aggregate. Whereas the term liposome refers to an artificial membrane construction derived from amphiphiles which form a bilayer in an aqueous medium, the oleosome is found in plant cells and consists of a self-enclosed single-layer membrane and an internal volume filled with triacylglycerols [47].

Being a two-dimensional structure with limited diffusion, the lipid bilayer is an inherently ideal system for concentrating components of signalling and metabolism. Thus, membranes often work as “key processors” of cellular information. Cell membranes are highly dynamic and deformable structures; they can be curved, tubular or flat ones, which results in distinct biophysical properties [68]. At the points of contact (contact sites), membranes from neighbouring organelles come together into a unique three-dimensional structure, forming functionally distinct microdomains that provide spatially regulated functions such as organelle communication. The shape, three-dimensional architecture and remodelling of the contact sites co-define their function in the cell [69].

Membrane contact sites can be determined as areas of membrane close localisation between different organelles. There are also examples of such contacts between membranes inside organelles. At the contact points of the membranes of two organelles, these membranes combine into a unique three-dimensional structure, forming functionally different microdomains that provide spatially regulated functions, such as organelle interaction. A considerable diversity in the geometry of the membranes forming the contact sites has been observed in various eukaryotic organisms. A physical interaction between organelles is provided by supramolecular protein assemblies that fill the intermembrane space and create binding forces through protein–protein or lipid–protein interactions. Membranes work by concentrating important elements at strategic points in the cell, thus acting as information processing centres. Activity in these centres requires bidirectional information processing across membranes and also between the neighbouring organelle membranes at membrane contact points [69].

Two parameters of cell membranes, their curvature and lipid profile, guide the selection of many peripheral proteins in cellular organelles. Although these features are often studied independently from each other, it is the combination of both that creates the unique interphase properties of biological membranes and, in particular, modulates the adhesive properties of membranes. The correlation between the separation of model amphipathic helices in membranes and the distribution of lipid packing defects in lipid bilayers of different shapes and compositions explains how macroscopic membrane properties modulate protein selection by altering the molecular topography of the lipid bilayer interphase region. One of the factors that should accentuate the defects in lipid packing in the membranes of the early secretory pathway is distortion. In general, defects in lipid packing may be beneficial for several functions of early secretory organelles and, in particular, biosynthetic processes. It has been suggested that the free packing of lipids in the ER promotes the folding of transmembrane proteins, whereas increased levels of CHOL or saturated PhL promote the accumulation of unfolded transmembrane proteins. Most of the ER consists of wispy (30 nm in radius) tubules, and the transport pathways between the ER and cis-Golgi also include the formation of small transport vesicles with the same radius. The bending of the lipid bilayer necessarily causes its lipids to tilt, an effect that should help to expose the hydrophobic cavities [70].

## 3. The Composition of Lipids in the Membranes of Various Eukaryotic Cell Organelles

Only PL—phospho- and glycolipids, as well as low-polarity lipids such as diacyl- and monoacylglycerols—are usually present in biological membranes. Within each of the rather extensive PhL and glycolipid groups there are subgroups of sphingolipids—sphingophospholipids [45,46] and sphingoglycolipids [32]. Plant membranes also have a class of phytoglycolipids that include elements of PhL, glyco- and sphingo-lipids in their molecules. It should be noted that we do not include free sterols and sterol glycosides in the total lipids and instead consider them as a separate group of compounds. A number of papers classify these compounds as lipids, probably according to their similar hydrophobicity to lipids. Sterols are a subgroup of steroids with a characteristic configuration consisting of four rings of carbon atoms. We propose that only those compounds containing residues of higher FA connected with polar radicals by ester or amide bonds should be considered as lipids [47]. In total, membrane lipids account for up to 500 types of these substances [20], or tens of thousands of individual compounds belonging to these types [71,72].

The set of polar lipids composing the bilayer matrix of cell membranes is very complex. It is not clear why there is such large diversity when most of the membrane functions, such as barrier properties, permeability and catalytic activity of internal membrane proteins, can be provided by applying a mixture of only a few molecularly determined lipids. Our knowledge on the chemical pathways operating to create and maintain the specific lipid profile of each morphologically individual membrane in eukaryotic cells is incomplete [73].

Most of the membrane PL are arranged in a similar way having a polar head and hydrophobic tails. Nevertheless, cells detect lipids with exceptional specificity. The functionality of lipids is defined by their local concentration, which differs between organelles, between two monolayers of the membrane and even in the lateral plane of the lipid bilayer. In order to determine their function, it is necessary not only to know which lipids are present at each specific intracellular location, but also their concentration, time period and interaction partners [71].

Glycerophospholipids (GPhL), sphingophospholipids represented by SM and also non-lipid CHOL serve as typical chiral molecules that constitute biological membranes. The combination of chiral lipids and CHOL creates a platform for functional cell membranes capable of regulating membrane receptor functions and enzymatic responses that are realized at the chiral membrane surface and in monolayers. Interlipid interactions in membranes occur in a stereoselective manner and induce the creation of the lipid phase and domains with different sizes ranging from tens of micrometres to nanometres in model lipid bilayers. It is known that some membrane proteins interact specifically with lipids and chiral ligands, while simultaneous interactions with surrounding lipids result in the regulation of protein function and localization [74].

Many cell membranes are known to be often asymmetric, i.e., the layers differ in the lipid profile. Thus, the outer layer may contain mainly phosphatidylcholines (PC) and glycosfingolipids, whereas the inner one may contain phosphatidylethanolamines (PE) and phosphatidylserines (PS), with phosphatidylinositol (PI) being found in both monolayers. The outer monolayer tends to contain PL with larger polar heads, as there is more surface area per polar head. The specific PhL of the mitochondrial internal membrane is cardiolipin (DPG); it is synthesised by mitochondrial inner membrane enzymes and represents about 22% of all PhL of this membrane [75]. DPG has a tendency to create a non-lamellar configuration that reduces the mechanical stability of the membrane; it is found in membranes generating electrical potential [47].

In some membranes, lipids can migrate from one side of the membrane to the other, i.e., to make “flip-flop” jumps. This movement of lipid molecules is hindered by their polar heads and so PL located on the internal side of the lipid bilayer have an advantage in this respect and have a relatively high rate of transmembrane motion. The most abundant lipids in eukaryotic membranes, such as PC and SM, have a polar head group for which the effective cross-section is larger than that of the two alkyl chains. Their head group lies almost parallel to the lipid bilayer surface and rotates rapidly. A large phosphocholine headgroup size is observed when the PC and SM molecules are strongly tilted in the gel state [76].

While the most quantitatively abundant PL—PC and PE, appear to create the matrix of biomembranes, a number of classes of lipids related to either charged (PG, DPG) or highly polar (phytoglycolipids) compounds with these two properties (PI, PS and sulphoquinovosyldiacylglycerol (SQDG)) compose lipid rafts. Integral proteins are important components of the membranes; they permeate the membranes and are responsible for a variety of their features. Next to membrane enzymes there are annular lipids; the latter are more ordered, less mobile, have more saturated FA in their structure and are extracted from membranes along with proteins. Most membrane proteins do not function without annular lipids [47].

Two classes of GPhL, namely phosphatidylglycerol (PG) and phosphatidylinositol (PI), are both the key structural components of the lipid membrane. They play a significant role in signal transduction, cell division and interaction with proteins. PG and PI are anionic lipids because of the presence of negatively charged functional groups. While the PG has one negatively charged phosphate group, the phosphate in the head group and the phosphate modification on the PI inositol ring can vary from one to three groups. The negative charge is important for these lipids to interact with the positive charge of membrane proteins; it plays a signalling or regulatory role in cellular metabolism [77].

Lipid head groups can have an excess charge, or can, conversely, be zwitterionic. The most prominent anionic lipids of cell membranes are the aforementioned PG as well as phosphatidylserine (PS). PS has a negatively charged phosphate group, a positively charged amino group and a negatively charged carboxyl group in the zone close to the hydrophobic group [78,79,80].

PI participates in membrane deformation, intracellular transport, the control of the organisation and dynamics of the actin cytoskeleton and the regulation of intracellular signalling pathways. The process of phosphorylation of the PI inosite ring is important in the expression of these functions. Phosphorylated PI becomes a multivalent negatively charged lipid; PI, PI-phosphate and PI-diphosphate are phosphorylated at one, two and three positions, respectively. The latter are of certain interest from the point of electrostatics due to having many charges per surface area of the molecule in the membrane [78].

Di- and triphosphoinositides represent a minor class of regulatory PhL; they participate in the regulation of membrane transport and cytoskeletal dynamics in eukaryotes [81,82]. It is important to note that phosphoinositides can bind various protein partners on the cytosolic surface of the PM, thereby influencing different processes [83]. PIs can perform an “anchoring” function in the membrane; they can be connected to specific proteins of the external surface of the membrane via an oligosaccharide. PI with an oligosaccharide (glycan) attached to one of the inositol hydroxyl groups is called phosphatidylinositglycan (PI-glycan). Proteins bind to this molecule via phosphoethanolamine; the protein can create a covalent bond with PI-glycan [47].

PC and sphingolipids are localised in the exoplasmic leaflet. Some glycosylsphingolipids, such as galactosylceramides, are primarily present in basolateral membranes while glucosylceramides are predominantly detected in apical membranes [9]. Experiments on the delivery of synthesised sphingolipids to epithelial cells have shown that a simple glycosylceramide, glycosylsphingolipid, was mainly transported to the apical membrane [41].

A characteristic property of the outer and inner membranes of chloroplasts is their high content of glycolipids as well as one of the phospholipids (PhL), phosphatidylglycerol (PG). Glycolipids account for 75% of all membrane lipids in chloroplast thylakoid membranes; the PG content in chloroplast membranes reaches 11% of the total lipids. The PG of chloroplast membranes includes a special FA, trans-3-hexadecenoic (16:3^Δ7,10,13^). Its presence is essential for the assembly of the light-harvesting complexes of chloroplasts [26].

There are considerably more glycolipids than PhL in the leaf tissues of plants. These glycolipids include monogalactosyldiacylglycerol (MGDG), digalactosyldiacylglycerol (DGDG) and the sulfolipid SQDG. Glycolipids play a main role in the biogenesis of thylakoid membranes and are important for the correct functioning of embedded photosynthetic pigment–protein complexes in plant cells. The main PhL of chloroplasts is PG.

In addition to structural variety, different classes of membrane lipids are not distributed evenly between organelles, membranes or even between two monolayers of the same membrane. Rather, they have special locations, and the cooperative action of their three-dimensional molecules determines the identity and feature of the various organelles. Thus, for example, galactolipids are located exclusively in the chloroplasts in plants, whereas the PM lipid microdomains are enriched in sterols and sphingolipids [2].

MGDG and DGDG are the main lipid classes in the photosynthetic membranes of chloroplasts. These lipids also serve as major components of the inner membrane structures called prolamellar bodies (PLBs) and prothylakoids (PTs) in etioplasts, which are formed in the seed cells of dark-growing, angiospermic plants. After being exposed to light, PLBs and PTs in etioplasts are converted into a photosynthetic membrane, leading to chloroplast development. During the differentiation of the etioplast into a chloroplast, galactolipids contribute to the formation of thylakoid membranes from PLBs and PTs; they play a key role in chlorophyll biosynthesis and in the accumulation of light-harvesting proteins [60]. Thylakoid membrane lipids, such as MGDG and PE, tend to create an inverted hexagonal phase as a result of their conical shape; so, they are called non-bilayer or non-lamella-forming lipids. Lipids with a cylindrical shape, PG, PC, DGDG and SQDG, create a lamellar phase and may be referred to as plate-forming or bilayer lipids [84].

One group of sphingoglycolipids that are analogous to SM in plant membranes, ceramides, have a long saturated N-acyl chain and a small polar headgroup, which makes them a lipid with very dense packaging properties. It is not surprising that ceramides can form rafts in model membranes and stabilize them. Individual C_18:0_ ceramide as well as mixtures of natural ceramides have the property to displace sterols from rafts [49].

Polar carotenoids and their acetates are also generally incorporated into the structure of lipid membranes and have their polar groups connected to the opposite polar zones of the bilayer. The pigment molecules provide rigidity to the liquid phase of the lipid bilayer and limit oxygen penetration into the hydrophobic core of the membrane vulnerable to oxidative damage due to the van der Waals interactions of the rigid rod-shaped carotenoid molecules and the acyl chains of the lipids. The presence of polar carotenoids in the lipid bilayer affects the physical properties of the membrane by changing membrane fluidity and altering the barrier of penetration for small molecules, especially oxygen [85].

The specific PhL of the internal mitochondrial membrane is cardiolipin (DPG), which accounts for over 20% of all PhL of this membrane. DPG has a negative charge; unlike the other acidic PhL (PS, PG, PI, and SQDG), it has two acid groups instead of having one group. The presence of DPG in artificial liposomes has been shown to result in an 8-fold increase in ribonuclease protein embedding into these liposomes compared to similar liposomes with PC. The positively charged ribonuclease prefers acidic PhL here [47].

Thus, both qualitative and quantitative compositions of PL in each individual organelle have an individual character. In addition, these indicators vary quite a lot in plants of different types, genera and in individual species. However, for planning experiments on modelling the membranes of different organelles, it is possible to use averaged data on the composition of the main PL components of these organelles [5,86,87].

## 4. Lateral Heterogeneity of Eukaryotic Cell Membranes

The lateral heterogeneity of eukaryotic cell membranes is a crucial parameter for the regulation of biological functions. Various physiological processes require the assembly and mutual organisation of specific membrane components. Lateral compositional and physico-chemical heterogeneity is a common property of cell membranes at different length scales, from small groups of molecules to micrometre domains. Separated lipid domains of increased local order called lipid rafts are thought to be characteristic of eukaryotic PM; however, their precise nature (composition, size, time of existence) in living cells remains difficult to determine [88,89,90].

The best known concept of membrane organisation, the lipid raft theory, associates the separation of the lipid phase (due to interactions between CHOL, sphingolipids and saturated PhL) with the separation and regulation of membrane proteins [41,72]. The study of lipid phase separation in membranes is an extremely interesting subject. A practical starting point is to represent the lipid bilayer as a two-dimensional (2D) fluid medium with various membrane components diffusing freely through it. Triple model membranes, including two types of PhL and CHOL at low temperature, separate macroscopically in phase into liquid-ordered (Lo) and liquid-disordered (Ld) domains. The same separation into two phases was observed in vesicles derived from the plasma membrane [17].

One of the most important properties of cell membranes is their mosaic nature, which is expressed by the constant presence of lateral nonhomogeneities, the sizes and lifetimes of which vary over a wide range from 1 to 100 nm and from 0.1 ns to milliseconds. While macroscopic domains are relatively well understood, the study of micro- and nanoclusters (or domains) that form a snapshot of the apportionment of dynamic, structural, electrical, hydrophobic, etc., properties at the membrane-water interface is of increasing interest. Many studies have proven that such nanodomains are very important for the proper functioning of cell membranes [9,43,53,88,91,92].

In addition to sphingoglycolipids and CHOL, lateral heterogeneities may include specific membrane proteins anchored to glycosylphosphatidylinositol (GPI). Lipid rafts comprising these components determine the sorting of related proteins and provide the assembly sites for cell-signalling molecules. These domains are probably less than 70 nm in diameter and are degraded when the cellular CHOL is removed. This confirms that lipid-bound proteins are organised into CHOL-dependent domains of submicron size [32].

The main arguments for rafts in cells are based on the observation that certain membrane proteins, including GPI anchored proteins and lipid bound non-receptor tyrosine kinases, glycosphingolipids and CHOL create detergent insoluble complexes in cold Triton X-100. The existence of such complexes is often seen as a proof for the presence of domains in the lipid bilayer. However, the presence of these complexes does not fully confirm the existence of lipid rafts in membranes. The functional features of GPI-bound proteins provide the most convincing arguments for the lateral separation of these proteins. Yet, there is another view that GPI-anchored proteins in eukaryotes are a various set of exoplasmic proteins diffusely dispersed across the cell surface, which contradicts the existence of rafts [32].

Biological membranes are structured and complex fluids; they are highly dynamic systems. They support fluctuating, cooperative and correlated dynamic regimes that lead to local order instead of disorder. At the same time, the lateral and transversal molecular organisation and local order of the membrane are related to the out-of-plane membrane architecture, resulting in lipid bilayer distortion, thickness changes, geometric shape transformations and the formation of non-lamellar phases [12].

The lipids and proteins that constitute biological membranes are located asymmetrically between the two sides of the membrane; they are also non-randomly dispersed on the sides in the membrane structure. Transmembrane asymmetry is caused by the manner of membrane synthesis and differentiation, as well as the effect of ATP-dependent lipid translocations. Lateral asymmetry in cell membranes can be divided into four main types. The first type are ordered structures in which membrane proteins are primarily responsible for the organisation of the structure. The second are the liquid membrane domains, where the interactions between internal membrane proteins and lipids that usually form non-bilayer structures result in the packaging of oligomeric complexes sealed within the bilayer matrix.

**Figure 1:**
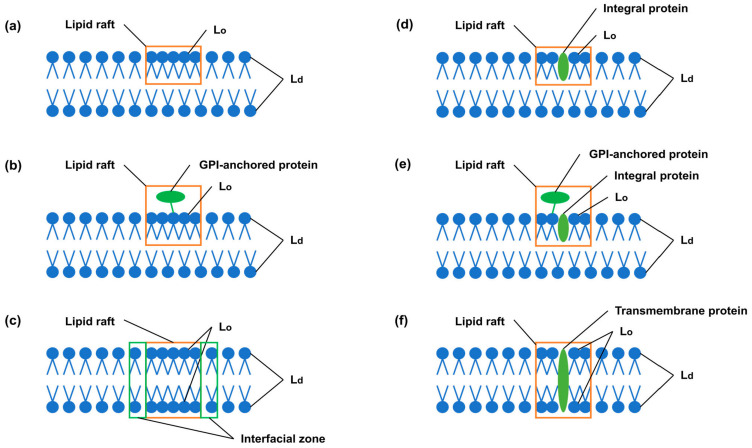
Types of lipid rafts in membranes. Lipid rafts include (**a**) the Lo zone in one monolayer of the membrane; (**b**) the Lo zone in one monolayer and GPI-anchored protein bound to membrane phosphatidylinositol; (**c**) the Lo zone in both monolayers opposite each other; (**d**) the Lo zone in one monolayer around the integral protein; (**e**) the Lo zone in one monolayer around the integral protein and GPI-anchored protein bound to membrane phosphatidylinositol; (**f**) the Lo zone around the transmembrane protein in both monolayers opposite each other. Lo and Ld—liquid-ordered and liquid-disordered lipid phases in membranes, respectively. The interfacial zone buffers a significant difference in ordering between Lo and Ld zones.

The third type are the so-called liquid-ordered structures resulting from the interaction of sterols [93] with membrane PLs. At present, there is disagreement about the size and exact composition of the liquid-ordered structure domains, but they are considered to be the main components of membrane rafts (Figure 1). Cholesterol associations with membrane lipids generate a discrete lipid compartment or domain with unique physical and biological properties. The cholesterol confers an ordering of the lipids that imparts changes to the physical properties of the membrane. The Lo domain can be formed in one monolayer and in both monolayers of the membrane. It is assumed that lipid rafts are able to include only those membrane proteins that are necessary for their functioning [88]. A lipid raft may include integral proteins localized in one monolayer, transmembrane proteins and GPI- anchored proteins bound to membrane phosphatidylinositol. An interfacial zone is formed around the lipid raft, which buffers a significant difference in ordering between the raft and non-raft domains. Rafts of less than 200 nm in size can form a framework that ensures the coordination of protein functions [94]. Finally, the fourth type is the formation of stoichiometric complexes between membrane lipids and CHOL, which assemble into a quasicrystal structure [88].

The local presence of calcium is known to cause mesoscopic changes in membrane organisation and geometry. A lateral reorganisation of the lipid bilayer in response to the presence of calcium ions is possible. When calcium ions were applied to vesicles enriched with negatively charged lipids, the tubular protrusions were formed. The formation of the negative curvature is shown as a result of the clustering of PI-diphosphate and PS anionic lipids in the presence of calcium ions [95]. 

The domain configuration is known to be formed by differences in the physico-chemical properties of membrane lipids, such as phase transition temperature, intermolecular hydrogen bonds and ionic functional groups. Domains are also formed by specific interactions between various membrane lipids and free sterols to create stoichiometric complexes (membrane rafts). The present task is to determine the balance of linear tension between lateral membrane domains in individual monolayers of the bilayer and the coupling forces acting on the middle plane of the bilayer, which is responsible for the maintenance of lipids in the opposite domains of the different monolayers of the membrane bilayer [88].

## 5. Cytoskeleton and Actin Networks

Membranes can be considered as a mosaic of regions supported by a cytoskeleton network. The cortical cytoskeleton has also been determined as a major player influencing the formation of membrane domains [9,17]. The cytoskeleton is a dense fibrous network of actin and spectrin on the cytoplasmic side of the cell plasma membrane. This network is coupled to the lipid bilayer via anchoring sites such as lipid-binding proteins, transmembrane proteins or membrane-attached proteins. Dense membrane-bound actin networks have been shown to have a major influence on lipid phase separation. This is how the hypothesis of the lateral compartmentalisation of the membrane—the “picket fence” model—emerged. According to this model, the lipid bilayer is divided into small compartments as a result of its interaction with a dense network of actin fibres, which acts as a skeleton inside the eukaryotic cell [17,96]. This mesh construction then acts as a barrier, limiting both lipid and protein diffusion and the macroscopic phase separation of lipids. A system proposed by the authors extends the ‘picket fence’ model by including a link between the local curvature of the membrane with its contents; it allows us to observe Lo domain formation in the presence of a lipid-linked actin network [17].

Thus, the most basic and largest membrane domain is the ‘membrane compartment’, which is created by the separation of the entire plasma membrane through its interaction with the actin-based membrane scaffold (“fencing”) and the transmembrane proteins attached to the membrane scaffolding (“pickets”) [34]. The data indicate that transmembrane proteins and the actin-containing cortical cytoskeleton can organise lipids into short-lived nanoscale groups, which can be assembled into larger domains under specific conditions [97]. Raft formation remains a hot topic in the field of membrane biology. A lot of information about raft structuring by the cortical actin cytoskeleton is known. It has previously been shown that the actin cytoskeleton is associated with lipid rafts and many structural and functional features of rafts require an intact actin cytoskeleton [98].

While actin-dependent mechanisms are fairly well known in animal cells, they are still poorly described in plant cells. Nevertheless, interactions and cross-links between microtubules and microfilaments have been found in plant cells, revealing the role of microtubule organisation in regulating the mobility of plant proteins. It has also been shown that the cellulose deposition pattern in the cell wall strongly affects the protein trajectory and diffusion velocity, indicating that the cell wall is involved in regulating the lateral organization of the plant plasma membrane [15]. The formation of protein nanoclusters attached to the GPI (up to four molecules) is an active process in which both actin and myosin are involved, and these nanoclusters are circulated non-randomly into larger domains of up to 450 nm [98,99].

The emergence of an alternative hypothesis, called a fence model, to characterize the behaviour of heterogeneous diffusion has thrown the theory of nanodomains into doubt. The hypothesis was that the diffuse behaviour is mainly influenced by the membrane-proximal actin cytoskeleton. A hierarchical model for describing PM that included both lipid nanodomains and actin-derived nanodomains, as well as a group defined as oligomerised membrane proteins, was proposed. It is possible that all of these types of domains are present in PMs [53].

## 6. Methods for the Study of Membranes and Lipid Rafts

The introduction of more advanced analytical methods has now increased spatial resolution by ×5000, from ~1.0 μm to ~0.2 nm. A fluorescent photobleaching technique is generally used for a visual demonstration of the mobility of proteins and lipids in the membrane [10].

Many data on cellular content come from the study of fixed cells or tissue homogenates using methods such as liquid chromatography–mass spectrometry and immunoblotting. These methods can demonstrate the presence of molecules but do not provide real-time information on their localization or interactions with each other, limiting our knowledge on the functions of the studied molecules. For the real-time imaging of labelled molecules in living cells, a fluorescence microscopy is the preferred tool (Table 1). However, fluorescent tags are too big for small molecules such as FA, amino acids and CHOL. These problems highlight the urgent need to develop chemical imaging platforms to enable an in situ or in vivo analysis of the molecules. For this purpose, the rapid vibrational imaging of individual tissues and cells with stimulated Raman scattering microscopy is used. Oscillatory spectroscopy, based on spontaneous Raman scattering, is widely used for the label-free analysis of the chemical composition of tissues and cells. The Raman process, however, has a weak effect, limiting its application to the rapid chemical imaging of a living cell [100].

Electron tomography (ET) is a method of three-dimensional electron microscopy with which we can obtain dissected views of cells from any direction and quantify their structural parameters with nanometre resolution. This improved electron microscopy method is suitable for the characterisation of twisted membrane elements and cellular components smaller than 100 nm. Investigations of plant cells fixed by rapid freezing have extended our knowledge of the properties and functions of plant cells and organelles. The ability of correlative electron and light microscopy for molecular imaging can be integrated with ET in studies of plant cells and organelles. Cryofixation and ET are among the most notable advances in electron microscopy, although they cannot be applicable to all objects. ET with the cryofixation of samples in vitreous ice (cryo-ET) allowed us to resolve the structure of organelles at the sub-nanometre scale. High-pressure freezing followed by freezing is the most practical way of cryopreserving plant cell samples [120].

Many sophisticated imaging methods (Table 1), such as confocal microscopy, TIRF, FRET or multiphoton laser scanning microscopy (Table 1, p.11), have been created in recent decades to study the biology of membranes in living systems [23,90]. Methods such as FRAP, FCS and FRET, as well as single molecule tracking and photoactivation techniques have greatly enhanced our understanding of molecular movement behaviour in living cells [106]. Atomic force microscopy, near-field optical microscopy and faster resonance energy transfer can all, in principle, serve to visualize the lipid nanodomains directly [121]. The application of electron paramagnetic resonance, nuclear magnetic resonance, DSC and X-ray diffractometry made it possible to determine the influence of carotenoids on the dynamic and structural features of lipid bilayers [85].

A simple and affordable technique for measuring domain sizes below the optical resolution limit using the coupled MC-FRET method has been proposed (Table 1, p. 15). It was found that a domain radius is about 7.5–10 nm for distearoylphosphatidylcholine (DSPC)/palmitoylphosphatidylcholine (POPC)/CHOL and ∼5 nm for SM/POPC/CHOL. The identification of such nanodomains can be achieved using FRET and SANS methods (Table 1, p. 11 and 13), as well as by using electron spin resonance. By means of interferometric scattering microscopy (Table 1, p. 14), the nanodomains can be observed even in the absence of any external marks [54].

Fluorescence microscopy techniques with multiphoton excitation are discussed for planar membrane systems such as lipid leaflets at the air–water interface (known as Langmuir films). The non-linear fluorescence microscopy method provides information with spatial and temporal resolution using the fluorescence properties of specific fluorescent probes. The use of environmentally-sensitive probes such as Laurdan, for example, enables measurements with the generalised polarisation function of Laurdan, which, in turn, is sensitive to the local packing of lipids in the bilayer [108].

Important data about temporarily stable but mainly dynamic nanodomains have been received by means of biophysical techniques such as STED, FRET and interferometric scattering (Table 1, p. 1, 11 and 14). The sizes of the nanodomains observed ranged from 5 to 60 nm [104,114]. The consistent use of FRET and SANS methods has significantly reduced the uncertainty in the estimates of domain size for mixtures of dioleoylphosphatidylcholine (DOPC) and POPC with SM/CHOL. FRET data have demonstrated coexisting domains for both mixtures [9]. Efforts in improving techniques for determining the configuration of membrane proteins using spin-labelled EPR spectroscopy hold promise for expanding the application of spin-labelling in structural biology. Long-distance measurements (60–80 Å) between pairs of spin labels allow for the quantitative study of equilibrium dynamics and induced conformational changes in protein structure [118]. Although NMR (Table 1, p. 20) allows for the direct determination of the dynamics of membrane proteins, it has low sensitivity and restrictions due to the molecular mass of the proteins. In contrast, sensitivity and size are not limiting for probe-based spectroscopic methods, such as fluorescence and spin-labelling EPR, when proteins can be studied in a medium more similar to that of native membranes [118].

A large number of sophisticated methods, such as single particle tracking, fluorescence microscopy, FRET and ESR, have been applied in studies aiming to explain the function of individual components of lipid rafts [105]. Confocal fluorescence microscopy demonstrates a regime of coexistence of ordered, DPPC-rich and DLPC-rich liquid lamellar phases with an upper boundary at a constant molar fraction of CHOL [105]. An extensive series of ESR studies (Table 1, p. 19), with restored integral membrane proteins, have shown that a constant amount of lipids is limited in movement by a direct interaction with each protein, regardless of the total lipid content of the bilayer [116].

Computer modelling and two innovative methods, STED microscopy (Table 1, p. 1) and FCS (Table 1, p. 6), were used to study the features of the model lipid bilayer in the presence of a dense network of actin fibres in precise detail. Lo domain formation in the presence of a lipid-linked actin network was observed using these methods [17]. The MC-FRET method was later used for the same purpose (Table 1, p. 15) [63]. The authors provided an expansion of the ‘picket fence’ model to include a link between the local curvature of the membrane and its composition [17].

The various lipid phases can be visualised via fluorescence microscopy with tags that are allocated to the phases. This approach can be applied to micrometre-sized domains but is unsuitable for lipid rafts of 20–200 nm diameter because of the short dwell time of the tag within a particular raft and the achievable spatial resolution. The fluorescent probe can also disrupt the phase behaviour either directly or through photo-oxidation. In addition to fluorescence-based methods, a reflectance interference contrast microscopy is used to characterise phase segregation in lipid bilayers, which takes advantage of different bilayer thicknesses and refractive indices. This approach, assuming sufficient sensitivity and resolution, can be applied to domains as small as desired [101].

The combination of AFM (Table 1, p. 17) with high-resolution fluorescence microscopy is an appealing tool for determining membrane phases using both physical topography and fluorescence. This method has been used to study the capability of a set of fluorescent molecules to probe domain structures in the applied planar bilayers [122]. The AFM method can detect membrane microdomains using height differences between lipids existing in different phase modes, including rafts or Lo phases that are 0.5–1 nm higher than the encircling Ld phase in model membranes consisting of DOPC/SM/CHOL. The use of simultaneous AFM–confocal imaging has been applied to study the capability of different lipids such as ganglioside, PC, SM and CHOL to separate into Ld or Lo phases in planar lipid bilayers [122,123].

Two-dimensional lipid bilayers were investigated using spectroscopic imaging via the SERS method (Table 1, p. 10). The distereo-PC lipid bilayer incubated on a glass base was coated with a thin layer of silver. Due to the strong electromagnetic amplification of the silver film and the affinity to lipid molecules, the Raman spectrum of a single bilayer was obtained within an exposure time of 1 s at an incident laser power of 0.1 MW [111]. A combination of AFM (Table 1, p. 17) and near-field scanning optical microscopy techniques was applied to investigate phase segregation in membrane monolayers and bilayers on the substrate acting as models of membrane rafts. These methods are used for studying binary and ternary lipid mixtures, which have gel or Lo domains that range in size from tens of nanometres to tens of micrometres, surrounded by an Ld membrane phase [114].

With its high imaging speed and 3D spatial resolution, the CARS method (Table 1, p. 9) provides a new approach to the real-time vibrational imaging of individual cells and organelles in a living system [100]. This method is used to visualise giant vesicles of binary lipid mixtures. The vibrational selectivity of the CARS microscope allows molecules to be identified based on the differences in vibration modes [124].

The usage of deuterated PhL as one of the components of a binary mixture makes it possible to evaluate directly the structure of a particular component in the mixture throughout the phase separation region. Such PhLs serve to explore the practically non-perturbed components of a model membrane system. A platform that captures the temporal behaviour and structural organisation of macromolecular complexes in living cells is presented. Using the combination of live-cell partial wave spectroscopy (PWS) and quantitative imaging techniques, the measurements of cell dynamics have been combined with the macromolecular structure, creating a dual light interference platform (dual-PWS) that significantly improves the understanding of the physical state of the cell and investigates cell behaviour at the level of macromolecular junctions. Although dual-PWS is not molecularly specific, it reflects the basic behaviour of all macromolecular complexes [106].

## 7. Membrane and Lipid Raft Models

Due to the complexity of the cell membrane, scientists often use simpler membrane models and computer modelling to investigate how various types of lipid molecules are organised within the membrane [17,125,126].

The simplest model membranes are hydrated bilayers consisting of saturated and unsaturated PhL, such as dioleoylphosphatidylcholine (DOPC) and dipalmitoylphosphatidylcholine (DPPC). Their atomic structural details are difficult for experimental characterisation; however, by means of computer modelling of the molecular dynamics (MD) for all the atoms, the key information can be obtained. The lateral location of lipids in these systems reveals small geometric and hydrophobic clusters on the surface. While being “sharp” inside the bilayer, the lateral heterogeneity is very “blurred” at the surface. It presents a very fuzzy picture, as the lifetime of the clusters is only 1 ns. In a binary system, DPPC acts as an order preference agent, which effectively regulates the behaviour of the DOPC [58]. Langmuir films are used as planar membrane model systems, allowing the measurement of surface pressure, molecular area isotherms and surface potential isotherms [127].

The zwitterionic lipids PC, PE and SM, with head groups having two opposing elementary charges, are commonly used for the production of artificial membranes. Considerable progress has been achieved by investigating chemically simplified models of the external monolayer of mammalian membranes. Mixtures containing three lipid compounds—high-melting PhL (with high Tm content; di-saturated PC or SM), PhL with low Tm content (with one or two unsaturated chains) and CHOL—demonstrate the key features related to lipid rafts. These minimal systems simulate the composition of specific biological membranes and reconstitute many complex phenomena, including the coexistence of Lo and Ld phases (domains). Surprisingly, their compositional simplicity makes them suitable for studying composition- and temperature-dependent behaviour [50].

When modelling triple mixtures of SM/DOPC/CHOL and DPPC/DOPC/CHOL, it was detected that there were significant differences in the composition of the Lo and Ld domains formed by these two mixtures. The interaction of lipids and CHOL shows the special role of hydrogen bonds between the amide SM and CHOL. The SM amide plane is most suitable for a specific configuration of hydrogen bonds, which changes the local hexagonal order in the Lo domain compared to a mixture with DPPC [52].

Since the molecular profile of cell membranes is highly varied, the inherent features of lipid rafts have been thoroughly investigated in simplified model membranes of ternary lipid compositions where the raft-imitating Lo phase coexists with the non-raft Ld phase. The Lo phase was found to consist mainly of saturated lipids with a high melting point, while CHOL in this simple system has many properties of lipid rafts in cell membranes [2].

Many studies have, for a long time, been focused on PM and especially on its external monolayer. Based on the lipid content of the latter, the composition of lipids in the three-component lipid mixtures in the model experiments on membranes was selected. At present, the membranes of all cell organelles require the same detailed study, keeping in mind the composition of the major lipid classes in each of these membranes, especially since there are many methods for studying these model membranes. The same requirements must be considered when studying lateral heterogeneity, lipid rafts, Lo and Ld phases. Not only CHOL, SM and some randomly taken PhL should be taken into account, but also the specific lipid profile of each individual membrane of any organelle.

It is obvious that in the simulation experiments using CHOL and SM alone, no membrane will be created, and the Lo and Ld phases will not be formed. A third component is needed at least, i.e., an unsaturated PhL, which is the only one that can create the Ld phase. However, PhLs in living organisms are not only unsaturated as they come in three types, also including saturated and semi-saturated forms. Thus, the model system must consist of the three types of the mentioned PhL in addition to CHOL and SM. The resulting lipid rafts will also contain saturated PC among the usual components for rafts. Semi-saturated PC is likely to be found at the interface between Lo and Ld [128] or to “dilute” both of these phases. In the large-scale all-atom MD experiments performed with the model membrane, a narrow (2 nm wide) interfacial zone of palmitoyl-docosahexaenoyl-PC, SM and CHOL was observed to be formed around the raft-like domain, which buffers a significant difference in order between the raft-like and non-raft-like media [128]. In the case of CHOL, it is naturally predominantly located in the lipid raft or in the Lo phase, since unsaturated PhLs (and possibly semi-saturated PhLs) have a significant repulsive effect on it in the Ld phase [129]. Nevertheless, there are reports that CHOL is divided between Lo and Ld phases in about equal parts and that there is no strict preference for any of these domains [130].

Currently, scientists have used a computer modelling approach and two advanced methods—STED microscopy (Table 1, p. 1) and FCS spectroscopy (Table 1, p. 6)—to study the features of the model lipid bilayer in the presence of a dense network of actin fibres. The results demonstrate that in accordance with the predictions of the ‘picket fence’ model, the membrane-bound actin fibres prevent the separation of the lipid phase at low temperatures. Furthermore, actin fibres also help to organise the distribution of lipids and proteins within the lipid bilayer at physiological temperatures. It is suggested that actin fibres cause the membrane to bend in a way that may increase the impact of the “picket fence”. The results showed that the “raft” and “picket fence” models are linked and the cell can control the interactions between the lipid bilayer and the actin fibres which make up the part of cytoskeleton [17,102].

The model membranes proved to be a valuable system to study hydrophobic compliance. The “mattress model” foretells that the insertion of a rigid helical transmembrane protein into a liquid bilayer leads to the local deformation of the bilayer. Adaptive lipid bending and straightening can also be accompanied by a tilt of the protein. This selective association of the corresponding lipids with transmembrane proteins, which causes a bilayer deformation, has been predicted by both theory and modelling. This also applies to the macroscopic sorting processes according to hydrophobic length and the elasticity property of membranes modulated by CHOL [126].

There is a need for model systems with reduced difficulty that still sufficiently imitate PM. Currently, minimal model membrane systems, i.e., supported lipid bilayers (SLB), giant unilayer vesicles (GUV) and giant PM vesicles (GPMV), and their application in the investigation of protein–membrane interactions, are the most widely used ones [131]. The most common of these are giant unilayer vesicles (GUV), which are either lipid-only systems or additionally contain purified and recovered proteins. Lo and Ld phases can coexist in GUV membranes depending on the lipid composition. GUV membranes are usually made up of only a very small number of lipid and protein species; so, the lack of complex composition makes it difficult to compare the results obtained in vitro with the real situation in the cell [125]. The combination of GUV, two-photon fluorescence microscopy and a Laurdan probe was proven to be extremely useful to obtain a microscopic picture of the coexistence of lipid phases in a two-layer GUV model system. Laurdan is a unique probe that provides synchronous information on the morphology and the phase state of lipid domains via fluorescence imaging [103].

A medium model system between the fully synthetic GUV and living cell membranes is the GPMV, a microscopic PM sphere derived from living cells after chemical processing. GPMV resembles native biological membranes with regard to the diversity of lipids and proteins but has the disadvantage of being rather highly variable in composition and complexity [55]. The creation of ordered, selective, lipid-driven domains in the resulting GPMV membranes is an important piece of evidence for the possibility of forming these domains in living cells. There are several crucial differences between PMs in living cells and insulated GPMV, including the loss of strong membrane asymmetry and degradation of some lipids during the creation of GPMV [55,125,131].

Several simple membrane models with coexisting Lo and Ld lipid phases have been generated to simulate the heterogeneous organisation of cell membranes; this helps to study the nature and functional role of ordered lipid–protein nanodomains named “rafts”. Despite their much reduced complexity, the quantification of local lipid media by using model lipid bilayers is not trivial, and the parallels to be drawn with cell membranes are not always apparent. At the same time, various, fluorescent-labelled lipid analogues have been used to investigate the membrane organisation and properties in vitro, although the biological activity of these probes with respect to their native analogues often remains uncharacterised, since the molecular architecture of free sterols and lipids is susceptible to disruption via fluorescent labelling [55].

To visualise the distribution of SM in the lipid rafts using Raman microscopy, an SM analogue labelled with a Raman-active diyne fragment (diyne-SM) was synthesised. Raman microscopy was used for the direct visualisation of the diyne-SM distribution in the raft, imitating domains created in triple SM/DOPC/CHOL monolayers. Raman images visualised an inhomogeneous diyne-SM distribution, which exhibited noticeable variations even within a single ordered region [56]. In particular, the diyne-SM content in the central region of the raft domains was significantly higher than that in the peripheral region. These data seem inconsistent with the mainly accepted raft model, where the raft and non-raft domains exhibit a clear two-phase separation. One possible reason is that the gradual changes in SM concentration occur between SM-rich and SM-poor areas to minimise hydrophobic mismatch [56].

Reducing the proportion of CHOL led to the solubilisation of model lipid rafts, whereas adding CHOL led to an increase in the size of SM-rich domains and eventually to the creation of a single lipid raft-like phase. The affinity of CHOL for SM-rich domains was confirmed by using the sterol-binding agent filipin [17,132]. In the CHOL–sphingolipid raft model, the latter are associated laterally with each other, probably due to weak interactions between the carbohydrate heads of the glycosphingolipids. The sphingolipid head groups occupy more space in an exoplasmic monolayer than do the saturated PhL. Any gaps between the associated sphingolipids are filled with CHOL molecules and they function as spacers [41].

A further interpretation of the biochemical reactions occurring in the cell would be not be possible without studying the physical and chemical properties of the lipid bilayer and, very importantly, the structure of membranes at the molecular level. It can be assumed that individual PL, sterols, peripheral and integral proteins and some other compounds are contained in membranes in certain molar ratios. Thus, one of the available ways to investigate the structure of these aggregates may be to establish such a ratio, i.e., to determine the molar concentration of each PL class and all other components of an individual membrane reliably and precisely and to build a theoretical model of the membrane based on the results obtained. Despite the significant advances in the biophysics of model systems used for the study of biological membranes, considerable problems have arisen; currently, these include the lack of data linked to the complete analysis of the lipid profile in each organelle or in a specific section of the lipid bilayer. Performing such studies is difficult sometimes due to the considerable complexity of the qualitative composition of lipids in plant cells [47].

As presented by many authors, the composition of PLs in individual membranes, so far, is hardly involved further in the study of the structure and functioning of these membranes. To overcome the conceptual and technical barriers for the further study of the structure of eukaryotic membranes, an interdisciplinary approach involving the collaboration of biochemists, physicists, mathematicians and computer scientists is needed [5].

## 8. Computer Modelling of Membrane Structure

Eukaryotic membranes are difficult to study. They are complex in terms of lipid composition and structure, operate over an extensive range of timescales and they are characterised by non-equilibrium conditions. The application of modern scanning electron microscopes (SEM) with a spatial resolution of 3 nm [133] and a high-resolution transmission electron microscopes (HRTEM) with a spatial resolution of 0.05–0.2 nm [134] has provided significant progress in the study of cell membranes. Despite the achievements in the experimental methods for directly exploring living cells, the detailed organisation of membranes appears to be too complicated to study at the molecular level. Therefore, modelling is an excellent method for studying the behaviour of biomembranes [1,57,58,59,135,136]. A substantial part of the functional processes in eukaryotic membranes proceeds at the molecular level; thus, computational modelling is the technique of choice for studying how their properties arise from specific molecular features and how interactions between multiple molecules lead to functioning at spatial and temporal scales that exceed the molecular ones. Methods such as molecular dynamics (MD) simulation are able to describe the interactions between all the components of a system with atomic resolution, acting as a ‘computational microscope’ [136,137]. Sufficient computer power enables the system’s behaviour to be monitored over a prolonged period of time in order to observe the process of interest [1,59].

The numerous structures and functions such as the actin-cytoskeleton network, the glycocalyx network and non-equilibrium transport under ATP-driven conditions have been, so far, given little attention; however, the modelling potential to solve these problems is extraordinarily high. Probably, future studies will show that computer modelling does investigate cell membranes, not just lipid bilayers [1]. Thanks to the steady increase in computing power caused by the efficient use of GPUs, as well as the development of accurate atomistic and coarse-grained models and the development of community-based tools for the automatisation of the setup and analysis of membrane modelling, we are now seeing a shift from simplistic to multi-component, realistic membrane modelling. Such a switch is useful to elucidate lipid–protein interactions in the very complex environment of real eukaryotic membranes, where experimental measurements are difficult to obtain, and theoretical models fall short of expectations [59].

The MD modelling method has become a crucial tool in structural biology. It can provide knowledge on atomic-scale processes that are often unavailable using current experimental methods; so, atomistic modelling is often used to supplement experimental investigations. MD modelling can also provide important information on large-scale behaviour, such as phase segregation and diffusion, when simplified (coarse-grained) molecular models are used instead of atomistic models [61,138]. The use of MD and IXS modelling techniques (Table 1, p. 18) is in fact a nanoscale method of the direct investigation of energy transfer and collective short-wave dynamics in biologically relevant model bilayers [115].

Considering the complexity of the biological membrane, scientists often use simpler membrane models and computational modelling to investigate how various types of lipid molecules are organised within the lipid bilayer. In accordance with the “picket fence” model, the biological membrane is separated into small compartments as a result of its interaction with a dense network of actin fibres, which acts as a cytoskeleton within the living cell [17].

The progress of atomistic simulation models has now reached a level where the computational modelling is an important addition to experimental studies. Thus, an increase in computational resources has made millisecond atomistic modelling available; this is an important moment, as the functioning of many membrane receptors takes place exactly at a millisecond time scale [1].

Several biochemical and biophysical questions concerning the study of biomembranes have become possible to solve through modelling, using a relatively free computational resources. Leadership-level machines provide access to more sophisticated, by several orders of magnitude, research questions. Coarse-grained models allow us to conduct complex studies with computational costs that are 2–3 orders of magnitude lower than those of the analogous atomistic models [46,137].

The universal and widely used molecular modelling software program CHARMM (Chemistry in Harvard Molecular Mechanics) is well known. This program focuses on important molecules, such as nucleic acids, lipids, carbohydrates, proteins and low-molecular-weight ligands. For the study of these molecules, the program proposes a large list of computer tools, such as methods of sampling conformations and trajectories, free energy calculations, methods of molecular minimization, dynamics and analysis, as well as the capabilities of building models. CHARMM is a multifunctional and customizable molecular simulation and modelling computer program which uses classical and quantum mechanical energy functions for molecular systems of many various sizes, classes and levels of heterogeneity and difficulty. Information for calculations with proteins, lipids, nucleic acids and carbohydrates is available in a separate part of the program. CHARMM provides a wide range of analysis tools; they include comparison of static structure and energy, time series, correlation functions and statistical properties of molecular dynamic trajectories, as well as interfaces to computer graphics programs. CHARMM has been installed on many various machines and platforms and has been adapted to work efficiently on many types of computer systems, ranging from single-processor PCs, Mac and Linux workstations, vector or multicore machines, Linux clusters with distributed memory and large supercomputer installations with shared memory [135].

In 2022, Abbasi et al. presented a program product written in Python programming language, called CellSys, which can greatly simplify the construction of biological membrane structures for applications in cell systems. The program is designed for computer theoretical studies applying molecular modelling methods related to research in the field of drug development and drug delivery to cells. CellSys allows for generating new knowledge about the interactions of drug molecules with biological membranes. The program is used to manipulate data for generating structural models of cell membranes to provide important information for research in drug delivery systems Additionally, CellSys can be used for the long-term storage of data on membrane structures that will be used in the creation of new drugs and their delivery systems [139].

It should be noted that the LIPID MAPS consortium has created a series of bioinformatic tools for the creation of ‘on-demand’ lipid structures based on stored lipid patterns. The consortium presents the LIPID MAPS Structure Database (LMSD), where all the lipid molecules are annotated and classified, applying the nomenclature created by the LIPID MAPS consortium. LIPID MAPS laboratories are involved in the authentication, description and quantitative analysis of known and unknown lipids applying mass spectrometry (MS) and liquid chromatography (LC) experimental methods. Data about different lipid standards produced for these experiments, together with the protocols used, are presented on the Lipidomics Gateway website. This internet resource contains a set of structure drawing tools for several lipid types, such as glycerolipids, glycerophospholipids, sterols, fatty acyls, sphingolipids, cardiolipins and sphingolipid glycans. The consortium has developed a suite of easy online interfaces that allow the end user to quickly generate diverse chemical structures of lipids, as well as relevant systematic names and ontological information [140].

**Figure 2:**
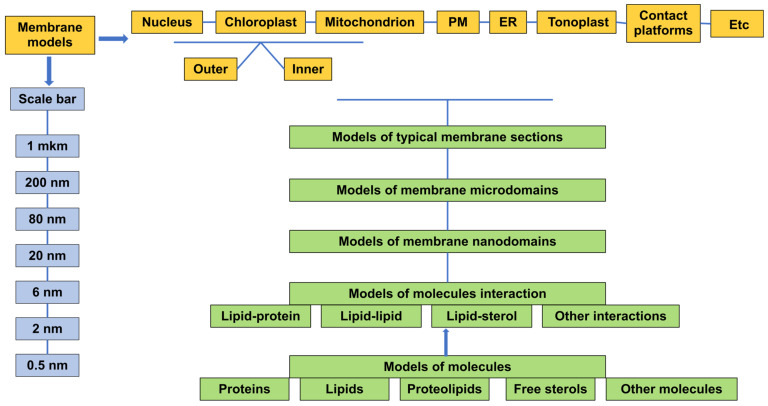
A possible scheme of the “encyclopaedia of biomembranes”. The database should contain information about models of all biological membranes, including the outer and inner membranes of organelles (nucleus, chloroplast, mitochondria), as well as their individual sites with special properties (contact platforms). For each membrane, models of typical membrane sections, microdomains, nanodomains, molecules of biomembrane components, as well as models of interaction of molecules at the scales from 1 μm to 0.5 nm should be presented. PM: plasma membrane; ER: endoplasmic reticulum.

Experimental research on the structure of biomembranes is currently being supplemented in several parallel ways: through the creation of simplified membrane models from ready-made chemical compounds; through a computer modelling approach, where new findings are generated in the electronic brain; through the creation of theoretical models of membranes or parts of them. All of these produce good results, but the extensive data obtained are often difficult to access for new researchers. We propose the creation of a platform that would be more accessible, garnering the quintessence of the vast amount of experimental data and serving as a source for new experiments, a place for the findings and an incentive for further research in this area. This should be a new computer database (library) on eukaryotic cell membranes, an example of which is the already existing Kyoto Encyclopedia of Genes and Genomes (KEGG), launched in Japan. There are similar programmes—CHARMM, LIPID MAPS—but they do not match the role we are assigning to the new platform. This platform or database should include information on the membrane models of all existing eukaryotic organelles (including PM), each of the double membranes, as well as their individual characteristic sites (Figure 2). There should not only be models of membranes and their sites, but also three-dimensional models of PL molecules, proteins, sterols and other compounds that make up biological membranes. This also applies to models of the tertiary and quaternary structure of protein molecules. These studies must take into account the composition of the major PL classes in each individual membrane studied. Finally, data on the interactions of the listed individual compounds with each other and three-dimensional models of these possible complexes should be included in this database.

Models introduced in the proposed library should be given at several scales, if possible. A computer platform could include, for example, seven types of images of each individual membrane at the scale where 1 cm corresponds to 1 μm, 200 nm, 80 nm, 20 nm, 6 nm, 2 nm and 0.5 nm. For example, the hydrogen bond size may be 0.27 nm.

## 9. Conclusions 

The study of eukaryotic membranes is one of the most prospective areas of modern biology; it occupies an important place in the research of the life cycle of the cell. An understanding of the structural organisation and functioning of biomembranes will make it possible to influence them in a targeted way in the future and to control the individual links involved in cell metabolism. The study of plant membranes is noticeably behind in comparison to the advances made in animal and microbial cells in terms of this important area of research. Therefore, in this work, we gave priority to the membranes of plant cells.

When studying membranes, it is important to understand that PM is not the standard structure of all other membranes. The membranes of eukaryotic organisms separate the cell into individual subcellular compartments that carry out crucial, but often non-compatible, metabolic reactions. The organelles are differentiated both quantitatively and qualitatively from the point of lipid content. The membranes of chloroplasts and mitochondria, for example, have a lipid composition that differs sharply from that of PM. Animal tissues differ from plant tissues in PL composition, and the internal and external membranes of the two-membrane organelles are also different.

The ways of interaction of actin and myosin with cell membranes require further study. Theoretical models of the “picket fence” type must find experimental validation. A computer simulation involving the cytoskeleton and actin networks can also help.

Nowadays, the opportunities of computer and computational techniques will probably allow for the construction of spatial, three-dimensional membrane models and, taking into account the advances in stereochemistry, the study of protein–lipid contacts, interactions of sterols with surrounding lipids and other compounds, the interaction of enzymes with their lipid microenvironment, and the dense packing of chlorophyll and other porphyrins in membranes.

The methods of computational modelling are a powerful tool for solving problems that are beyond the reach of current experimental methods and act as a “computational microscope”. A number of such methods, e.g., MD, are able to describe the interactions between all compounds of a system with atomic resolution. In the field of biomolecular computer modelling, the progress of atomistic simulation models has achieved a level where it has become a significant addition to experimental studies. Moreover, the increase in computational resources has made millisecond atomistic modelling available, which is particularly important when the same time scale of activation of many membrane receptors is taken into account [1]. Having the sufficient power of a computer, the process can be monitored for quite a long time while clusters containing only a few lipid molecules can be detected [141]. The significant expansion of the available area with a wide range of applications has led to an explosion in the use of modelling for the study of biomembranes [46].

It can be expected that the creation of an “encyclopaedia of membranes” will stimulate new research in this field and help to intensify the study of membrane structure. Advances can also be expected in the modelling of the molecules of those compounds which make up membranes and the possible situations of modelling the contacts of such molecules to each other. The construction of new three-dimensional physico-chemical and stereochemical models of many characteristic membranes or their specific sites will simplify the investigation of the roles of PL, proteins and sterols in performing their multiple functions in the membranes.

A further understanding of the biochemical reactions occurring in the cell would not be possible without studying the physical and chemical properties of the lipid bilayer and, very importantly, the structure of membranes at the molecular level. Being a two-dimensional structure with limited diffusion, the lipid bilayer is an inherently ideal system for concentrating components of signalling and metabolism. Membranes and membrane proteins allow the cell to interact with the external environment. Thus, membranes often work as “key processors” of cellular information. The so-called “computational microscope”—a simulation of PL molecular dynamics—is likely to serve as a major tool for studying their effects on the structure and function of biomembrane proteins. Further progress in the study of membrane properties requires significant improvements in the analytical methods and computational modelling used, as well as an interdisciplinary approach involving the collaboration of chemists, biophysicists and computer scientists.

## Figures and Tables

**Table 1:** Some methods used to study the structure of eukaryotic membranes.

№	Methods for Studying the Structure of Membranes	Reference
1.	Super-resolution optical STED microscopy (with stimulated emission depletion)	[17,101,102]
2.	Fluorescence-labelled microscopy	[52,101,103,104]
3.	Total internal reflectance fluorescence (TIRF)	[23,90]
4.	Confocal fluorescent microscopy	[23,90,105]
5.	Fluorescence recovery after photobleaching (FRAP)	[97,106]
6.	Fluorescence correlation spectroscopy (FCS)	[17,102,106,107]
7.	Reflectance interference contrast microscopy (in addition to fluorescence-based methods)	[101]
8.	Fluorescence microscopy with multiphoton excitation (using LAURDAN probes)	[108]
9.	Coherent anti-Stokes Raman light scattering (CARS) microscopy	[56,100,109,110]
10.	Spectroscopic imaging using surface enhanced Raman spectroscopy (SERS)	[111,112]
11.	Multiphoton laser scanning microscopy (with Förster resonance energy transfer analysis (FRET)	[9,32,50,63,90,96,97,104,106,113]
12.	Fluorescent photo-bleaching	[10]
13.	Consistent use of FRET and small-angle neutron scattering (SANS)	[9]
14.	Interferometric scattering microscopy	[54,62]
15.	Analysis of data obtained by FRET with Monte Carlo modelling (MC-FRET)	[54,63]
16.	Differential scanning calorimetry (DSC)	[9,50]
17.	Combination of atomic force microscopy (AFM) and near-field scanning optical microscopy	[114]
18.	Inelastic X-ray scattering (IXS)	[115]
19.	Electron spin resonance with spin label (ESR)	[116]
20.	Nuclear magnetic resonance (NMR)	[117,118]
21.	X-ray crystallography	[118]
22.	Spin-labelled EPR spectroscopy	[117]
23.	Two-photon microscopy	[119]
24.	Electron Paramagnetic Resonance (EPR)	[85]
25.	Oscillatory spectroscopy of spontaneous Raman scattering	[100]

## Data Availability

Data are contained within the article.

## References

[1] Enkavi G., Javanainen M., Kulig W., Rog T., Vattulainen I. (2019). Multiscale simulations of biological membranes: The challenge to understand biological phenomena in a living substance. Chem. Rev..

[2] Yu L., Zhou C., Fan J., Shanklin J., Xu C. (2021). Mechanisms and functions of membrane lipid remodeling in plants. Plant J..

[3] Engelman D.M. (2005). Membranes are more mosaic than fluid. Nature.

[4] Higashi S., Fujimura Y., Murata N., Quinn P.J., Harwood J.L. (1990). Analysis of lipids in spinach photosystem 2. Plant Lipid Biochemistry, Structure and Utilization.

[5] Van Meer G., Voelker D.R., Feigenson G.W. (2008). Membrane lipids: Where they are and how they behave. Nat. Rev. Mol. Cell Biol..

[6] Dowhan W., Bogdanov M., Vance D.E., Vance J.E. (2002). Functional roles of lipids in membranes. Biochemistry of Lipids, Lipoproteins and Membranes.

[7] Epand R.M. (2015). Introduction to membrane lipids. Methods Mol. Biol..

[8] Schmid K.M., Ohlrogge J.B., Vance D.E., Vance J.E. (2002). Lipid metabolism in plants. Biochemistry of Lipids, Lipoproteins and Membranes.

[9] Efremov R.G. (2021). Dinamic “molecular portraits” of biomembranes drawn by their lateral nanoscale inhomogeneities. Int. J. Mol. Sci..

[10] Watson H. (2015). Biological membranes. Essays Biochem..

[11] Singer S.J., Nicolson G.L. (1972). The fluid mosaic model of the structure of cell membranes. Science.

[12] Gil T., Ipsen J.H., Mouritsen O.G., Sabra M.C., Sperotto M.M., Zuckermann M.J. (1998). Theoretical analysis of protein organization in lipid membranes. Biochim. Biophys. Acta.

[13] Nicolson G.L. (2014). The Fluid—Mosaic Model of Membrane Structure: Still relevant to understanding the structure, function and dynamics of biological membranes after more than 40 years. Biochim. Biophys. Acta.

[14] Cacas J.L., Furt F., Le Guedard M., Schmitter J.M., Bure C., Gerbeau-Pissot P., Mongrand S. (2012). Lipids of plant membrane rafts. Prog. Lipid Res..

[15] Grosjean K., Der C., Robert F., Thomas D., Mongrand S., Simon-Plas F., Gerbeau-Pissot P. (2018). Interactions between lipids and proteins are critical for organization of plasma membrane-ordered domains in tobacco BY-2 cells. J. Exp. Bot..

[16] Bagatolli L., Mouritsen O. (2013). Is the fluid mosaic (and the accompanying raft hypothesis) a suitable model to describe fundamental features of biological membranes? What may be missing?. Front. Plant Sci..

[17] Honigmann A., Sadeghi S., Keller J., Hell S.W., Eggeling C., Vink R. (2014). A lipid bound actin meshwork organizes liquid phase separation in model membranes. Elife.

[18] Piao J., Yuan W., Dong Y. (2021). Recent progress of DNA nanostructures on amphiphilic membranes. Macromol. Biosci..

[19] Klotzsch E., Schuetz G.J. (2012). A critical survey of methods to detect plasma membrane rafts. Philos. Trans. R. Soc. B Biol. Sci..

[20] Bagatolli L.A., Ipsen J.H., Simonsen A.C., Mouritsen O.G. (2010). An outlook on the organization of lipids in membranes: Searching for a realistic connection with the organization of biological membranes. Prog. Lipid Res..

[21] Sibold J., Kettelhoit K., Vuong L., Liu F., Werz D.B., Steinem C. (2019). Synthesis of Gb_3_ glycosphingolipids with labeled head groups: Distribution in phase-separated giant unilamellar vesicles. Angew. Chem. Int. Ed. Engl..

[22] Ahmed S.N., Brown D.A., London E. (1997). On the origin of sphingolipid/cholesterol-rich detergent-insoluble cell membranes: Physiological concentration of cholesterol and sphingolipid induce formation of a detergent-insoluble, liquid-ordered lipid phase in model membranes. Biochemistry.

[23] Diaz M.L. (2010). Membrane physiology and the biophysics in the next decade: An open balcony to multiple scenarios. Front. Physiol..

[24] Troncoso-Ponce M.A., Nikovics K., Chloe M. (2016). New insights on the organization and regulation of the fatty acid biosynthetic network in the model higher plant Arabidopsis thaliana. Biochimie.

[25] Staehelin L.A., Paolillo D.J. (2020). A brief history of how microscopic studies led to the elucidation of the 3D architecture and macromolecular organization of higher plant thylakoids. Photosynth. Res..

[26] Raven J.A. (2021). Determinants, and implications, of the shape and size of thylakoids and cristae. J. Plant Physiol..

[27] Cassim A.M., Gouguet P., Gronnier J., Laurent N., Germain V., Grison M., Boutte Y., Gerbeau-Pissot P., Simon-Plass F., Mongrand S. (2019). Plant lipids: Key players of plasma membrane organization and function. Prog. Lipid Res..

[28] Ozolina N.V., Kapustina I.S., Gurina V.V., Nurminsky V.N. (2022). Role of tonoplast microdomains in plant cell protection against osmotic stress. Planta.

[29] Moller I.M., Rasmusson A.G., Van Aken O. (2021). Plant Mitochondria–Past, present and future. Plant J..

[30] Zang J., Kriechbaumer V., Wang P. (2021). Plant cytoskeletons and the endoplasmic reticulum network organization. J. Plant Physiol..

[31] Gronnier J., Legrand A., Loquet A., Habenstein B., Germain V., Mongrand S. (2019). Mechanisms governing subcompartmentalization of biological membranes. Curr. Opin. Plant Biol..

[32] Varma R., Mayor S. (1998). GPI-anchored proteins are organized in submicron domains at the cell surface. Nature.

[33] Klymchenko A.S., Kreder R. (2014). Fluorescent probes for lipid rafts: From model membranes to living cells. Chem. Biol..

[34] Kusumi A., Suzuki K.G., Kasai R.S., Ritchie K., Fujiwara T.K. (2011). Hierarchical mesoscale domain organization of the plasma membrane. Trends Biochem. Sci..

[35] Sengupta P., Baird B., Holowka D. (2007). Lipid rafts, fluid/fluid phase separation, and their relevance to plasma membrane structure and function. Semin. Cell Dev. Biol..

[36] Simons K. (2010). Lipid rafts as a membrane-organizing principle. Science.

[37] Sezgin E., Levental I., Mayor S., Eggeling C. (2017). The mystery of membrane organization: Composition, regulation and roles of lipid rafts. Nat. Rev. Mol. Cell Biol..

[38] Goni F.M. (2019). “Rafts”: A nickname for putative transient nanodomains. Chem. Phys. Lipids.

[39] Regen S.L. (2020). The Origin of Lipid Rafts. Biochemistry.

[40] Wang C., Krause M.R., Regen S.L. (2015). Push and pull forces in lipid raft formation: The push can be as important as the pull. J. Am. Chem. Soc..

[41] Simons K., Ikonen E. (1997). Functional rafts in cell membranes. Nature.

[42] Tsai Y.T., Moore W., Kim H., Budin I. (2020). Bringing rafts to life: Lessons learned from lipid organization across diverse biological membranes. Chem. Phys. Lipids.

[43] Ozolina N.V., Nesterkina I.S., Gurina V.V., Nurminsky V.N. (2020). Non-detergent isolation of membrane structures from beet plasmalemma and tonoplast having lipid composition characteristic of rafts. J. Membr. Biol..

[44] Bocharov E.V., Mineev K.S., Pavlov K.V., Akimov S.A., Kuznetsov A.S., Efremov R.G., Arseniev A.S. (2017). Helix-helix interactions in membrane domains of bitopic proteins: Specificity and role of lipid environment. Biochim. Biophys. Acta Biomembr..

[45] Keyvanloo A., Shaghaghi M., Zuckermann M.J., Thewalt J.L. (2018). The phase behavior and organization of sphingomyelin/cholesterol membranes: A deuterium NMR study. Biophys. J..

[46] Pandit S.A., Jakobsson E., Scott H.L. (2004). Simulation of the early stages of nano-domain formation in mixed bilayers of sphingomyelin, cholesterol, and dioleylphosphatidylcholine. Biophys. J..

[47] Zhukov A.V. (2021). On qualitative composition of membrane lipids in plant cells. Russ. J. Plant Physiol..

[48] Rondelli V., Koutsioubas A., Prsic J., Deboever E., Crowet J.M., Lins L., Deleu M. (2021). Sitosterol and glucosylceramide cooperative transversal and lateral uneven distribution in plant membranes. Sci. Rep..

[49] Megha, Sawatzki P., Kolter T., Bittman R., London E. (2007). Effect of ceramide *N*-acyl chain and polar headgroup structure on the properties of ordered lipid domains (lipid rafts). Biochim. Biophys. Acta.

[50] Petruzielo R.S., Heberle F.A., Feigenson G.W. (2013). Phase behavior and domain size in sphingomyelin-containing lipid bilayers. Biochim. Biophys. Acta.

[51] Cebecauer M., Amaro M., Jurkiewicz P., Sarmento M.J., Sachl R., Cwiklik L., Hof M. (2018). Membrane lipid nanodomains. Chem. Rev..

[52] Sodt A.J., Pastor R.W., Lyman E. (2015). Hexagonal substructure and hydrogen bonding in liquid-ordered phases containing palmitoyl sphingomyelin. Biophys. J..

[53] Kure J.L., Andersen C.B., Mortensen K.I., Wiseman P.W., Arnspang E.C. (2020). Revealing plasma membrane nano-domains with diffusion analysis methods. Membranes.

[54] Enoki T.A., Heberle F.A., Feigenson G.W. (2018). FRET detects the size of nanodomains for coexisting liquid-disordered and liquid-ordered phases. Biophys. J..

[55] Sezgin E., Levental I., Grzybek M., Schwarzmann G., Mueller V., Honigmann A., Belov V.N., Eggeling C., Coskun U., Simons K. (2012). Partitioning, diffusion, and ligand binding of raft lipid analogs in model and cellular plasma membranes. Biochim. Biophys. Acta.

[56] Ando J., Kinoshita M., Cui J., Yamakoshi H., Dodo K., Fujita K., Murata M., Sodeoka M. (2015). Sphingomyelin distribution in lipid rafts of artificial monolayer membranes visualized by Raman microscopy. Proc. Natl. Acad. Sci. USA.

[57] Berger O., Edholm O., Jähnig F. (1997). Molecular dynamics simulations of a fluid bilayer of dipalmitoylphosphatidylcholine at full hydration, constant pressure, and constant temperature. Biophys. J..

[58] Pyrkova D.V., Tarasova N.K., Pyrkov T.V., Krylov N.A., Efremov R.G. (2011). Atomic-scale lateral heterogeneity and dynamics of two-component lipid bilayers composed of saturated and unsaturated phosphatidylcholines. Soft Matter.

[59] Marrink S.J., Corradi V., Souza P.C.T., Ingolfsson H.I., Tieleman D.P., Sansom M.S.P. (2019). Computational modeling of realistic cell membranes. Chem. Rev..

[60] Fujii S., Wada H., Kobayashi K. (2019). Role of galactolipids in plastid differentiation before and after light exposure. Plants.

[61] Rog T., Vattulainen I. (2014). Cholesterul, sphingolipids, and glycolipids: What do we know about their role ijn raft-like membranes?. Chem. Phys. Lipids.

[62] Li X., Gu Y. (2020). Structural and functional insight into the nuclear pore complex and nuclear transport receptors in plant stress signaling. Curr. Opin. Plant Biol..

[63] Sarmento M.J., Hof M., Sachl R. (2020). Interleaflet coupling of lipid nanodomains—Insights from in vitro systems. Front. Cell Dev. Biol..

[64] Ingolfsson H.I., Melo M.N., van Eerden F.J., Arnarez C., Lopez C.A., Wassenaar T.A., Periole X., de Vries A.H., Tieleman D.P., Marrink S.J. (2014). Lipid organization of the plasma membrane. J. Am. Chem. Soc..

[65] Kriechbaumer V., Brandizzi F. (2020). The plant endoplasmic reticulum: An organized chaos of tubules and sheets with multiple functions. J. Microsc..

[66] Theodoulou F.L., Carrier D.J., Schaedler T.A., Baldwin S.A., Baker A. (2016). How to move an amphipathic molecule across a lipid bilayer: Different mechanisms for different ABC transporters?. Biochem. Soc. Trans..

[67] Pan R., Liu J., Wang S., Hu J. (2020). Peroxisomes: Versatile organelles with diverse roles in plants. New Phytol..

[68] Mouritsen O.G. (2013). Physical chemistry of curvature and curvature stress in membranes. Curr. Phys. Chem..

[69] Rosado A., Bayer E.M. (2021). Geometry and cellular function of organelle membrane interfaces. Plant Physiol..

[70] Vanni S., Hirose H., Barelli H., Antonny B., Gautier R. (2014). A sub-nanometre view of how membrane curvature and composition modulate lipid packing and protein recruitment. Nat. Commun..

[71] Van Meer G. (2005). Cellular lipidomics. EMBO J..

[72] Simons K., Sampaio J.L. (2011). Membrane organization and lipid rafts. Cold Spring Harb. Perspect. Biol..

[73] Quinn P.J., Wolf C. (2009). The liquid-ordered phase in membranes. Biochim. Biophys. Acta.

[74] Hanashima S., Yano Y., Murata M. (2020). Enantiomers of phospholipids and cholesterol: A key to decipher lipid-lipid interplay in membrane. Chirality.

[75] Hsieh T.C.Y., Lester R.L., Laine R.A. (1981). Glycophosphoceramides from plants. Purification and characterization of a novel tetrasaccaride derived from tobacco leaf glycolipids. J. Biol. Chem..

[76] Somerharju P., Virtanen J.A., Cheng K.H. (1999). Lateral organization of membrane lipids. The superlattice view. Biochim. Biophys. Acta.

[77] Xia T., Ren H., Zhang W., Xia Y. (2020). Lipidome-wide characterization of phosphatidylinositols and phosphatidylglycerols on CC location level. Anal. Chim. Acta.

[78] Bohinc K., Spadina M., Rescic J., Shimokawa N., Spada S. (2022). Influence of charge lipid head group structures on electric double layer properties. J. Chem. Theory Comput..

[79] Valentine M.L., Waterland M.K., Fathizadeh A., Elber R., Baiz C.R. (2021). Interfacial dynamics in lipid membranes: The effects of headgroup structures. J. Phys. Chem. B.

[80] Efremov R.G., Pyrkova D.V., Krylov N.A. (2018). Fine tuning of microscopic properties in two-component zwitterionic-anionic lipid bilayers: Determinant role of H-bonding. Biophys. J..

[81] Heilmann I., Ischebeck T. (2016). Male functions and malfunctions: The impact of phosphoinositides on pollen development and pollen tube growth. Plant Reprod..

[82] Keller H., Lorizate M., Schwille P. (2009). PI(4,5)P_2_ degradation promotes the formation of cytoskeleton-free model membrane systems. ChemPhysChem.

[83] Fratini M., Krishnamoorthy P., Stenzel I., Riechmann M., Matzner M., Bacia K., Heilmann M., Heilmann I. (2021). Plasma membrane nano-organization specifies phosphoinositide effects on Rho-GTPases and actin dynamics in tobacco pollen tubes. Plant Cell.

[84] Wilhelm C., Goss R., Garab G. (2020). The fluid-mosaic membrane theory in the context of photosynthetic membranes: Is the thylakoid membrane more like a mixed crystal or like a fluid?. J. Plant Physiol..

[85] Gruszecki W.I., Strzalka K. (2005). Carotenoids as modulators of lipid membrane physical properties. Biochim. Biophys. Acta.

[86] Murray W.C., Ansell G.V., Hawthorne J.N., Dawson R.M.C. (1973). Phospholipids in subcellular organelles and membranes. Form and Function of Phospholipids.

[87] Olsson M., Norberg P., Liljenberg C., Quinn P.J., Harwood J.L. (1990). Changes of the membrane lipid composition during ontogenetical ageing. Plant Lipid Biochemistry, Structure and Utilization.

[88] Quinn P.J. (2012). Lipid–lipid interactions in bilayer membranes: Married couples and casual liaisons. Prog. Lipid Res..

[89] Kinnun J.J., Bolmatov D., Lavrentovich M.O., Katsaras J. (2020). Lateral heterogeneity and domain formation in cellular membranes. Chem. Phys. Lipids.

[90] Lu S.M., Fairn G.D. (2018). Mesoscale organization of domains in the plasma membrane—Beyond the lipid raft. Crit. Rev. Biochem. Mol. Biol..

[91] Heerklotz H. (2002). Triton promotes domain formation in lipid raft mixtures. Biophys. J..

[92] Schmid F. (2017). Physical mechanisms of micro-and nanodomain formation in multicomponent lipid membranes. Biochim. Biophys. Acta Biomembr..

[93] Ivanova N., Genova J., Chamati H. (2021). Physical properties of SOPC lipid membranes containing cholesterol by molecular dynamics simulation. Adv. Biomembr. Lipid Self-Assem..

[94] Saitov A., Kalutsky M.A., Galimzyanov T.R., Glasnov T., Horner A., Akimov S.A., Pohl P. (2022). Determinants of lipid domain size. Int. J. Mol. Sci..

[95] Sahoo A., Matysiak S. (2020). Microscopic picture of calcium-assisted lipid demixing and membrane remodeling using multiscale simulations. J. Phys. Chem. B..

[96] Raghupathy R., Anilkumar A.A., Polley A., Singh P.P., Yadav M., Johnson C., Suryawanshi S., Saikam V., Sawant S.D., Panda A. (2015). Transbilayer lipid interactions mediate nanoclustering of lipid-anchored proteins. Cell.

[97] Neumann A.K., Itano M.S., Jacobson K. (2010). Understanding lipid rafts and other related membrane domains. F1000 Biol. Rep..

[98] Chichili G.R., Rodgers W. (2009). Cytoskeleton–membrane interactions in membrane raft structure. Cell. Mol. Life Sci..

[99] Friedrichson T., Kurzchalia T.V. (1998). Microdomains of GPI-anchored proteins in living cells revealed by crosslinking. Nature.

[100] Zhang D., Wang P., Slipchenko M.N., Cheng J.X. (2014). Fast vibrational imaging of single cells and tissues by stimulated Raman scattering microscopy. Acc. Chem. Res..

[101] De Wit G., Danial J.S., Kukura P., Wallace M.I. (2015). Dynamic label-free imaging of lipid nanodomains. Proc. Natl Acad. Sci. USA.

[102] Sarangi N.K., Basu J.K. (2019). Preferential binding and re-organization of nanoscale domains on model lipid membranes by pore-forming toxins: Insight from STED-FCS. J. Phys D Appl. Phys..

[103] Bagatolli L.A., Sanchez S.A., Hazlett T., Gratton E. (2003). Giant vesicles, Laurdan, and two-photon fluorescence microscopy: Evidence of lipid lateral separation in bilayers. Methods Enzymol..

[104] Grzybek M., Kozubek A., Dubielecka P., Sikorski A.F. (2005). Rafts-the current picture. Folia Histochem. Cytobiol..

[105] Feigenson G.W., Buboltz J.T. (2001). Ternary phase diagram of dipalmitoyl-PC/dilauroyl-PC/cholesterol: Nanoscopic domain formation driven by cholesterol. Biophys. J..

[106] Gladstein S., Almassalha L.M., Cherkezyan L., Chandler J.E., Eshein A., Eid A., Zhang D., Wu W., Bauer G.M., Stephens A.D. (2019). Multimodal interference-based imaging of nanoscale structure and macromolecular motion uncovers UV induced cellular paroxysm. Nat. Commun..

[107] Korlach J., Schwille P., Webb W.W., Feigenson G.W. (1999). Characterization of lipid bilayer phases by confocal microscopy and fluorescence correlation spectroscopy. Proc. Natl. Acad. Sci. USA.

[108] Brewer J., de la Serna J.B., Wagner K., Bagatolli L.A. (2010). Multiphoton excitation fluorescence microscopy in planar membrane systems. Biochim. Biophys. Acta.

[109] Kinoshita M., Suzuki K.G.N., Murata M., Matsumori N. (2018). Evidence of lipid rafts based on the partition and dynamic behavior of sphingomyelins. Chem. Phys. Lipids.

[110] Li L., Wang H., Cheng J.X. (2005). Quantitative coherent anti-Stokes Raman scattering imaging of lipid distribution in coexisting domains. Biophys. J..

[111] Sweetenham C.S., Notingher I. (2010). Raman spectroscopy methods for detecting and imaging supported lipid bilayers. Spectroscopy.

[112] Mendelson R., Koch C.C. (1980). Deuterated phospholipids as Raman spectroscopic probes of membrane structure. Phase diagrams for the dipalmitoyl phosphatidylcholine (and its d62 derivative)-dipalmitoyl phosphatidylethanolamine system. Biochim. Biophys. Acta.

[113] Yano Y., Hanashima S., Hiroshi T., Slotte J.P., London E., Murata M. (2020). Sphingomyelins and ent-sphingomyelins form homophilic nano-subdomains within liquid ordered domains. Biophys. J..

[114] Johnston L.J. (2007). Nanoscale imaging of domains in supported lipid membranes. Langmuir.

[115] Bolmatov D., Katsaras J., Soloviov D., Zhernenkov M., Suvorov A., Cai Y.Q., Zavyalov D., Mamontov E. (2020). Molecular picture of the transient nature of lipid rafts. Langmuir.

[116] Pali T., Bashtovyy D., Marsh D. (2006). Stoichiometry of lipid interactions with transmembrane proteins—Deduced from the 3D structures. Protein Sci..

[117] Mchaourab H.S., Steed P.R., Kazmier K. (2011). Toward the fourth dimension of membrane protein structure: Insights into dynamics from spin-labeling EPR spectroscopy. Structure.

[118] Marsh D. (2003). Lipid interactions with transmembrane proteins. Cell. Mol. Life Sci..

[119] Gaus K., Gratton E., Kable E.P., Jones A.S., Gelissen I., Kritharides L., Jessup W. (2003). Visualizing lipid structure and raft domains in living cells with two-photon microscopy. Proc. Natl. Acad. Sci. USA.

[120] Wang P., Liang Z., Kang B.H. (2019). Electron tomography of plant organelles and the outlook for correlative microscopic approaches. New Phytol..

[121] Eggeling C., Ringemann C., Medda R., Schwarzmann G., Sandhoff K., Polyakova S., Belov V.N., Hein B., Middendorff C., Schonle A. (2009). Direct observation of the nanoscale dynamics of membrane lipids in a living cell. Nature.

[122] Shaw J.E., Epand R.F., Epand R.M., Li Z., Bittman R., Yip C.M. (2006). Correlated fluorescence-atomic force microscopy of membrane domains: Structure of fluorescence probes determines lipid localization. Biophys. J..

[123] Yuan C., Furlong J., Burgos P., Johnston L.J. (2002). The size of lipid rafts: An atomic force microscopy study of ganglioside GM1 domains in sphingomyelin/DOPC/cholesterol membranes. Biophys. J..

[124] Potma E.O., Xie X.S. (2005). Direct visualization of lipid phase segregation in single lipid bilayers with coherent anti-Stokes Raman scattering microscopy. ChemPhysChem.

[125] Owen D.M., Williamson D.J., Magenau A., Gaus K. (2012). Sub-resolution lipid domains exist in the plasma membrane and regulate protein diffusion and distribution. Nat. Commun..

[126] Kaiser H.J., Orlowski A., Rog T., Nyholm T.K.M., Chai W., Lingwood T.F.D., Vattulainen I., Simons K. (2011). Lateral sorting in model membranes by cholesterol-mediated hydrophobic matching. Proc. Natl Acad. Sci. USA.

[127] Alberts B., Johnson A., Lewis J., Raff M., Roberts K., Walter P. (2002). Molecular Biology of the Cell.

[128] Canner S.W., Feller S.E., Wassall S.R. (2021). Molecular organization of a raft-like domain in a polyunsaturated phospholipid bilayer: A supervised machine learning analysis of molecular dynamics simulations. J. Phys. Chem. B.

[129] Yang J., Jin J., Li S. (2021). Role of polyunsaturated phospholipids in liquid-ordered and liquid-disordered phases. RSC Adv..

[130] Lindblom G., Oradd G. (2009). Lipid lateral diffusion and membrane heterogeneity. Biochim. Biophys. Acta.

[131] Sezgin E., Schwille P. (2012). Model membrane platforms to study protein–membrane interactions. Mol. Membr. Biol..

[132] Lawrence J.C., Saslowsky D.E., Edwardson J.M., Henderson R.M. (2003). Real-time analysis of the effects of cholesterol on lipid raft behavior using atomic force microscopy. Biophys. J..

[133] Okada T., Ogura T. (2017). High-resolution imaging of living mammalian cells bound by nanobeads-connected antibodies in a medium using scanning electron-assisted dielectric microscopy. Sci. Rep..

[134] Kisielowski C., Freitag B., Bischoff M., van Lin H., Lazar S., Knippels G., Tiemeijer P., van der Stam M., von Harrach S., Stekelenburg M. (2008). Detection of single atoms and buried defects in three dimensions by aberration-corrected electron microscope with 0.5-Å information limit. Microsc. Microanal..

[135] Brooks B.R., Brooks C.L., Mackerell A.D., Nilsson L., Petrella R.J., Roux B., Won Y., Archontis G., Bartels C., Boresch S. (2009). CHARMM: The biomolecular simulation program. J. Comput. Chem..

[136] Ingolfsson H.I., Arnarez C., Periole X., Marrink S.J. (2016). Computational ’Microscopy’ Of Cellular Membranes. J. Cell Sci..

[137] Javanainen M., Martinez-Seara H., Vattulainen I. (2017). Excessive aggregation of membrane proteins in the Martini model. PLoS ONE.

[138] Li X., Zhou S., Lin X. (2022). Molecular view on the impact of DHA molecules on the physical properties of a model cell membrane. J. Chem. Inf. Model.

[139] Abbasi A., Amjad-Iranagh S., Dabir B. (2022). CellSys: An open-source tool for building initial structures for bio-membranes and drug-delivery systems. J. Comput. Chem..

[140] Subramaniam S., Fahy E., Gupta S., Sud M., Byrnes R.W., Cotter D., Dinasarapu A.R., Maurya M.R. (2011). Bioinformatics and systems biology of the lipidome. Chem. Rev..

[141] Pyrkova D.V., Tarasova N.K., Krylov N.A., Nolde D.E., Efremov R.G. (2011). Lateral clustering of lipids in hydrated bilayers composed of dioleoylphosphatidylcholine and dipalmitoylphosphatidylcholine. Biochemistry.

